# A proposed role for neutrophil extracellular traps in cancer immunoediting

**DOI:** 10.3389/fimmu.2013.00048

**Published:** 2013-03-06

**Authors:** Sivan Berger-Achituv, Volker Brinkmann, Ulrike Abu Abed, Lars I. Kühn, Jonathan Ben-Ezra, Ronit Elhasid, Arturo Zychlinsky

**Affiliations:** ^1^Research Laboratory for Pediatric Hemato-Oncology, Department of Pediatric Hemato-Oncology, Dana Children’s Hospital, Tel Aviv Sourasky Medical CenterTel Aviv, Israel; ^2^Microscopy Core Facility, Max Planck Institute for Infection BiologyBerlin, Germany; ^3^Department of Cellular Microbiology, Max Planck Institute for Infection BiologyBerlin, Germany; ^4^Department of Pathology, Tel Aviv Sourasky Medical CenterTel Aviv, Israel; ^5^Sackler Faculty of Medicine, Tel Aviv UniversityTel Aviv, Israel

**Keywords:** cancer, cancer immunoediting, Ewing sarcoma, neutrophils, neutrophil extracellular traps

## Abstract

Upon activation, neutrophils release fibers composed of chromatin and neutrophil proteins termed neutrophil extracellular traps (NETs). NETs trap and kill microbes, activate dendritic cells and T cells, and are implicated in autoimmune and vascular diseases. Given the growing interest in the role of neutrophils in cancer immunoediting and the diverse function of NETs, we searched for NETs release by tumor-associated neutrophils (TANs). Using pediatric Ewing sarcoma (ES) as a model, we retrospectively examined histopathological material from diagnostic biopsies of eight patients (mean ± SD age of 11.5 ± 4.7 years). TANs were found in six patients and in two of those we identified NETs. These two patients presented with metastatic disease and despite entering complete remission after intensive chemotherapy had an early relapse. NETs were not identified in the diagnostic biopsies of two patients with localized disease and two with metastatic disease. This study is the first to show that TANs in ES are activated to make NETs, pointing to a possible role of NETs in cancer.

## INTRODUCTION

Upon activation, neutrophils release neutrophil extracellular traps (NETs), which are fibers composed of chromatin and neutrophil proteins. NETs are released via a novel form of cell death called NETosis (Brinkmann and Zychlinsky, 2012). NETosis requires the production of reactive oxygen species (ROS; Fuchs et al., 2007), myeloperoxidase (MPO; Metzler et al., 2011) and on the translocation of neutrophil elastase (NE) from azurophilic granules to the nucleus (Papayannopoulos et al., 2010). Eventually, neutrophils release NETs that trap and kill microbes, activate dendritic cells (DCs) and T cells, and are implicated in autoimmune and vascular diseases (Brinkmann and Zychlinsky, 2012).

We postulated that NETs also have a role in cancer. The immune system can identify and destroy nascent tumor cells in a process termed cancer immunosurveillance. However, the immune system can also promote tumor progression. Together, these host-protective and tumor-promoting actions of immunity are referred to as cancer immunoediting (Kim et al., 2007). To our knowledge, there are no data on the possible role of NETs in this process.

Ewing sarcoma (ES) is the second most common primary bone cancer that afflicts adolescents and young adults. Although advances in diagnosis, surgery, chemotherapy, and radiation have substantially improved the survival rate of patients with localized ES to nearly 70%, the long-term outcome for those with metastatic or recurrent disease remains poor (Potratz et al., 2012). The importance of the immune system in anti-tumorigenic reactions against ES cells was previously shown. Therapy-naive ES patients manifest an inflammatory microenvironment with a high expression of type 1-associated chemokines and infiltration of CD8^+^ T lymphocytes, expressing corresponding chemokine receptors. Patients with higher numbers of tumor-infiltrating CD8^+^ T lymphocytes demonstrated a significant overall survival benefit (Berghuis et al., 2011). Potential targets for immune recognition specific to ES are protein products of the gene fusion EWS-ETS (Meyer-Wentrup et al., 2005) and other tumor-associated antigens like the ganglioside antigen GD2 (Kailayangiri et al., 2012). ES cells were also shown to be sensitive to natural killer (NK) cells (Cho et al., 2010) and to tumor necrosis factor (TNF)-related apoptosis-inducing ligand (TRAIL), a member of the TNF superfamily with strong anti-tumor activity and minimal toxicity to most normal cells and tissues (Mitsiades et al., 2001). By contrast, several mechanisms of immune escape are evident. Bone marrow T cells with a regulatory phenotype T(reg) were found at significantly higher numbers in patients with primary metastatic ES compared with localized ES (Brinkrolf et al., 2009). In addition, complete or partial absence of human leukocyte antigen (HLA) class I expression was observed in 79% of ES tumors. Lung metastases consistently lacked HLA class I with sequential tumors, demonstrating a tendency toward decreased expression with disease progression (Berghuis et al., 2009).

Given the absence of data in the literature on the role of NETs in cancer immunoediting, we used histopathological material from diagnostic biopsies of pediatric ES patients to identify NETs in the tumor bed.

## MATERIALS AND METHODS

### PATIENTS

We aimed to examine histopathological material from diagnostic biopsies of all ES patients (*n* = 14), who were treated in our Pediatric Hemato-Oncology Department, Dana Children’s Hospital, Tel Aviv Sourasky Medical Center, Tel Aviv, Israel, between July 2009 and July 2011. The histopathological material was taken from diagnostic biopsies, before initiation of chemotherapy or radiotherapy. All patients were previously healthy with no background systemic diseases or immunodeficiency and were free of infection during removal of the biopsy samples. Three patients were diagnosed elsewhere and therefore their histopathological material was not available and in another three the remaining material was insufficient for NET analysis. Of the remaining eight patients, six were males and two were females with a mean ± SD age of 11.5 ± 4.7 years. Further data are summarized in **Table 1**. The study was approved by the Institutional Review Board of Tel Aviv Sourasky Medical Center, Tel Aviv, Israel.

**Table 1:** Clinical and histopathological data.

Pat. No.	M/F	At diagnosis			Diagnostic biopsy		Chemotherapy-induced necrosis*** (%)	Months from diagnosis	
		Age (years)*	Primary disease site	Lung/bone metastasis	Neutrophils	NETs		Follow-up	Relapse
1	M	17	Lt. Iliac bone	yes	yes	yes	100****	30	22
2	M	15	Rt. proximal femur	yes	yes	yes	99	33	27
3	F	3	Lt. scapula	no	yes	no	97–98	33	no
4	M	12	Lt. iliac bone	no	yes	no	95****	31	no
5	M	9	Lt. iliac bone	yes	yes	no	99****	23	no
6	M	8	L4 vertebrae	yes	yes	no	100	13	no
7	F	16	Rt. distal fibula	no	no	NA**	100	43	no
8	M	13	Rt. metatarsal bones	no	no	NA**	100	41	no

*M/F, male/female; NA, not applicable.*

* *Mean age±SD, 11.5±4.7 years*.

** *Given that neutrophils were not identified in the tumor bed, further investigation for NETs was not performed*.

*** *The percentage of chemotherapy-induced necrosis, which is used as a prognostic factor for patient outcome, is measured during definitive surgery (i.e., after six courses of neo-adjuvant chemotherapy)*.

**** *Also received radiotherapy according to protocol*.

### TREATMENT PROTOCOL

Newly diagnosed non-metastatic ES patients received the Children’s Oncology Group (COG) AEWS0031 regimen B (i.e., chemotherapy intensification with alternate courses given every 2 weeks; Womer et al., 2012). ES patients with lung metastasis at diagnosis were treated according to the European EURO-E.W.I.N.G. 99 protocol R2 arm [i.e., consolidation with high-dose chemotherapy (busulfan–melphalan) and peripheral blood stem cell rescue; Ladenstein et al., 2010].

### NETs ANALYSIS

Hematoxylin and eosin (HE) stained sections of the ES biopsies were analyzed by a board-certified pathologist (Jonathan Ben-Ezra) for the presence of tumor-associated neutrophils (TANs). When TANs were identified, additional six 5-μm-thick sections were cut from the same block and stained for the presence of NETs as previously described (Brinkmann et al., 2004). In short, paraffin sections were hydrated before antigen retrieval by heating at 37°C for 50 min with target retrieval solution, pH 9 (Dako, Hamburg, Germany). The sections were blocked with 1% bovine serum albumin (BSA), 3% donkey serum (Millipore GmbH, Schwalbach/Ts., Germany), 3% cold fish gelatin (Sigma-Aldrich Chemie GmbH, Steinheim, Germany), 0.05% Tween, and 0.025% Triton for 30 min at room temperature, and incubated with primary antibodies against CD99 (AbD Serotec, Düsseldorf, Germany) and MPO (Dako, Hamburg, Germany) overnight at 4°C in a humid chamber. After washing, donkey anti-mouse Cy3 and donkey anti-rabbit Alexa 488 (Dianova, Hamburg, Germany) were used as secondary antibodies. The sections were also stained with the bisbenzimid DNA dye “Hoechst” H33342 (Sigma-Aldrich Chemie GmbH, Steinheim, Germany) and Draq5 DNA dye (New England Biolabs GmbH, Frankfurt/M., Germany). Confocal images were captured using Leica software (TCS-SP, Leica, Mannheim, Germany).

## RESULTS

Clinical and histopathological data on the eight ES patients that were included in the study are provided in **Table 1**. NETs were identified in the diagnostic biopsies of 2/8 patients: a 17-year-old-male with a large ES mass of the left iliac bone, L5 vertebrae involvement, and multiple lung metastases (patient 1) and a 15-year-old-male with ES of the right proximal femur and suspected lung metastases (patient 2). Both were treated with the EURO-E.W.I.N.G. 99 protocol and after entering complete remission underwent consolidation therapy with autologous stem cell transplantation. Both had an early relapse, 12 and 18 months after stem cell transplantation (22 and 27 months after diagnosis, respectively). In comparison, NETs were not found in the biopsies of four patients: a 3-year-old female and a 12-year-old male with localized disease and 9-year-old and 8-year-old males with lung metastases at diagnosis. These four patients are free of disease 33, 31, 23, and 13 months after diagnosis, respectively. In another two patients with localized disease, TANs were not identified, thus further investigation for NETs was not performed.

**FIGURE 1:**
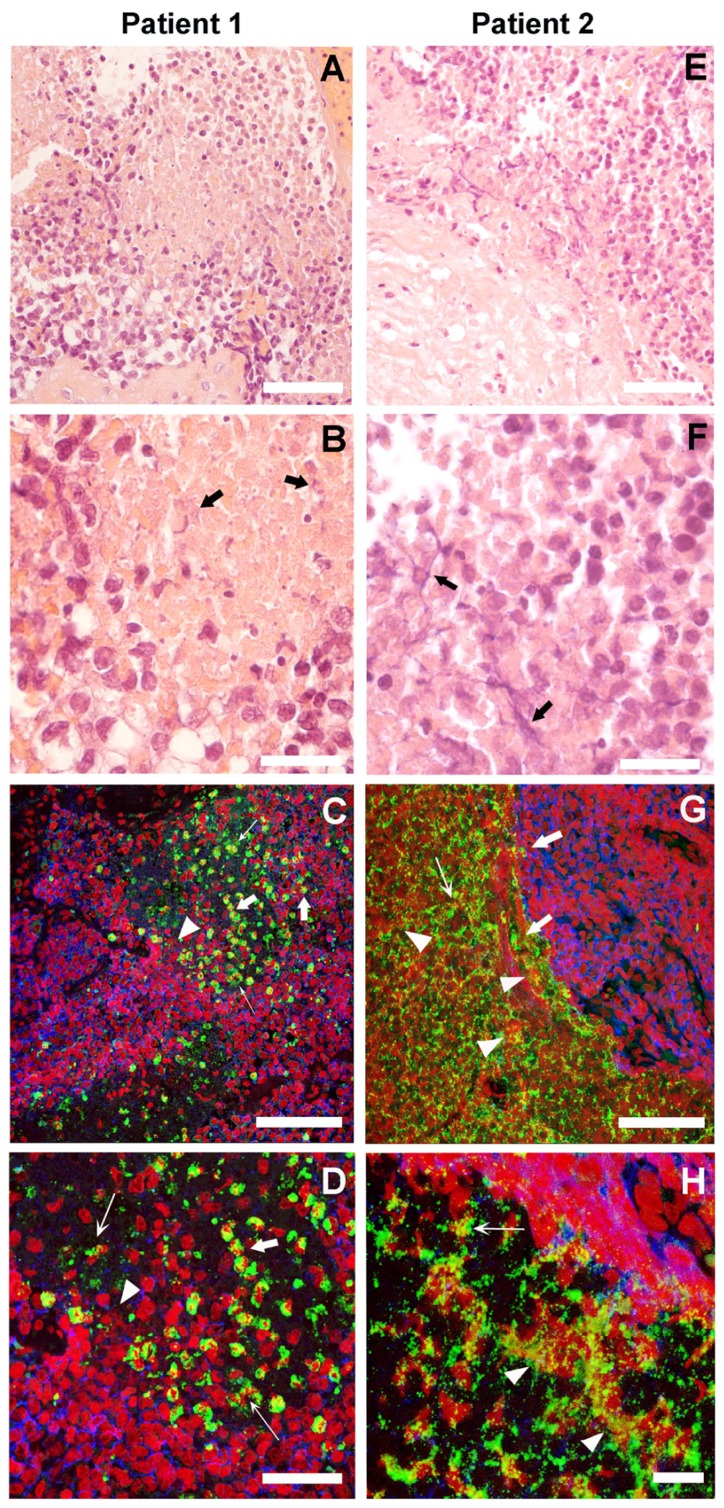
**Neutrophil extrcellular traps (NETs) in Ewing sarcoma**. The histological HE stained sections of two patients in low **(A,E)** and high **(B,F)** magnification suggest the presence of NETs (thick black arrows). Immunofluorescence staining for myeloperoxidase (MPO; green), CD99 (blue), and DNA (red) of sections adjacent to those shown in HE at low **(C,G)** and high **(D,H)** magnification shows projections of a z-stack of confocal sections 5-μm thick. Naïve Neutrophils are located where MPO is in granules (thick white arrows). Neutrophils in the process of making NETs are found where MPO is localized to delobulated nuclei (thin white arrows) and NETs are located where MPO is colocalized with extracellular DNA (white arrowheads; scale bars, 25 μm).

In the HE sections of both patients who demonstrated NETs (**Figures 1A,B,E,F**), we observed small round blue cells characteristic of ES. In patient 1, neutrophils invaded the ES cell mass; whereas in patient 2, neutrophils were adjacent to the ES cells inside, clearly defined necrotic tissue. In both patients, we observed material that is suggestive of NETs (thick black arrows). Adjacent sections with immunofluorescence staining demonstrated the presence of NETs (**Figures 1C,D,G,H**). In both patients, neutrophils were observed where MPO (green) is localized to the granules, indicating non-activated cells (thick white arrows). However, in many areas, MPO was localized to delobulated nuclei (thin white arrows) and to extracellular DNA stained in red (white arrowheads), attesting to the presence of NETs. In patient 1, NETs were formed in close contact with ES cells, which are labeled with CD99 antibodies (blue). Interestingly, CD99 appears degraded in areas where neutrophils are present. In patient 2, NETs were formed in the interface between ES cells and necrotic tissue. In the necrotic areas of both patients, we observed extracellular DNA that is not associated with MPO, indicating that these are not NETs.

## DISCUSSION

This study is the first to show that TANs in ES are activated to make NETs and thus points to a possible role for NETs in cancer. In the past, most of the research in the field of cancer immunoediting was confined to the role of T lymphocytes, NK cells, macrophages, and DCs (Kim et al., 2007); less attention was given to the role of neutrophils that are an essential effector cell of the innate immune system, serving as the first line of defense against infectious microorganisms. Neutrophils have either pro- or anti-tumor activity, depending on factors such as type of cancer and cytokine profile of the tumor microenvironment (Souto et al., 2011). For example, transforming growth factor (TGF)-β within the tumor induces a population of TANs with a pro-tumorigenic phenotype. However, in the absence of TGF-β, TANs are cytotoxic to tumor cells and express abundant pro-inflammatory cytokines (Fridlender et al., 2009). Neutrophils can promote tumor growth by secretion of matrix metalloproteinase (MMP)-9 that prevents tumor cell apoptosis in the lungs (Acuff et al., 2006) and can promote tumor angiogenesis and neovascularization (Masson et al., 2005). Nevertheless, neutrophils can also be cytotoxic to tumor cells by producing several types of ROS (Lichtenstein, 1987; Dallegri et al., 1991). Notably, in a mouse model of breast cancer, neutrophils were shown to inhibit metastatic seeding by generating hydrogen peroxide (Granot et al., 2011). Neutrophils also produce defensins, which can lyse cancer cells, recruit other immune cells, such as DCs, and have anti-angiogenetic properties (Al-Benna et al., 2011).

We speculate that NETs could have anti-tumorigenic effects, for example by actual killing of tumor cells or activating the immune system. Alternatively, NETs could have a pro-tumorigenic activity by facilitating metastases. Indeed, the three-dimensional structure of NETs may serve to physically capture tumor cells and prevent their dissemination to adjacent tissues. Several components of NETs have been shown to be cytotoxic to tumor cells. MPO was shown to kill B-16 melanoma cells and inhibit their growth in mice after implantation (Odajima et al., 1996). It is of note that MPO deficient patients may have an exceptionally high incidence of cancer (7/14 patients, 50%; Lanza et al., 1988). NETs can kill activated endothelial cell (Gupta et al., 2010), probably through histones (Saffarzadeh et al., 2012), damaging tumor-feeding blood vessels. NE secreted by TANs can cleave cyclin E (CCNE) to its low molecular weight isoforms and thus promote their presentation to cytotoxic T lymphocytes (Mittendorf et al., 2012). NETs modulate the link between innate and adaptive immune responses by activating plasmacytoid DCs through toll-like receptor 9 (TLR9), an intracellular receptor that recognizes DNA. This NET-mediated activation is important in autoimmune diseases like psoriasis (Skrzeczynska-Moncznik et al., 2012) and systemic lupus erythematosus (Lande et al., 2011). We postulate that tumor antigens caught on NET components could be displayed to DCs and cause their activation. NETs also prime T cells by TCR signaling that requires direct contact (Tillack et al., 2012). Alternatively, NETs, which harbor potent proteases, could be pro-tumorigenic by degradation of the extracellular matrix and promotion of metastases. NETs may also form a barrier between cancer cells and the immune system, thus assisting cancer cells to escape immune recognition. The fact that both our patients with metastatic disease and NET formation relapsed may point to the pro-tumorigenic mechanism of NETs.

Chemotherapy-induced necrosis is one of the most important predictors of outcome in non-metastatic ES patients. Less than 90% necrosis after neo-adjuvant chemotherapy predicts a higher relapse rate (Lin et al., 2007). Recently, van Maldegem et al. (2012) suggested the need for additional biomarkers to predict outcome and allow patient stratification into different risk groups. Our patients had a good histological response to chemotherapy including the two patients who relapsed (**Table 1**); therefore, the presence of NETs could possibly become an additional prognostic marker in ES and other cancers.

Our study is limited by its retrospective design. We examined biopsies that underwent standard histopathological processing and were not necessarily handled with the same care required for NETs preservation. Thus, we cannot rule out that NETs were not found in other samples because they were damaged during material handling or because only a small part of the tumor was sampled. The study is also limited by its small sample size, which precludes statistical analysis to firmly prove the clinical relevance of NET formation in ES. A larger prospective cohort of patients with diverse types of malignancies is advocated. However, we present these preliminary data due to the novelty of our finding, supporting the notion that NETs have a role in cancer immunoediting.

To conclude, this study provides the first data on the release of NETs by TANs in ES. Given that both patients with metastatic disease and NET formation relapsed may point to a pro-tumorigenic mechanism of NETs and may constitute an important prognostic marker in ES and in other cancers. This intriguing topic may have a significant contribution to our understanding of innate immune responses against cancer and potentially lead to the development of new therapeutic strategies in the battle against cancer.

## Conflict of Interest Statement

The authors declare that the research was conducted in the absence of any commercial or financial relationships that could be construed as a potential conflict of interest.
